# Examining the Effectiveness of Mothers and Babies Online Delivered in Home Visiting: A Pilot Randomized Controlled Trial

**DOI:** 10.21203/rs.3.rs-8991058/v1

**Published:** 2026-04-09

**Authors:** Darius Tandon, Jaime Hamil, Elaine McBride, Lara Baez, Blaire Pingeton, Bayley Taple, Alinne Barrera

**Affiliations:** Northwestern University; Northwestern University; Northwestern University; Northwestern University; Northwestern University; Northwestern University; Palo Alto University

**Keywords:** Postpartum depression, prevention, intervention, home visiting, online interventions

## Abstract

**Background:**

Although effective interventions exist to prevent postpartum depression, less evidence exists when examining online modalities. Mothers and Babies is an effective postpartum depression preventive intervention, with an online version developed to minimize barriers associated with in-person intervention delivery. Home visiting (HV) programs serve pregnant people and new mothers and provide an innovative setting for intervention delivery, given their ongoing service delivery with clients.

**Methods:**

Pregnant women and women with an infant under one year recruited from HV programs were randomized to an intervention or control condition. Intervention participants received an online version of the 7-lesson Mothers and Babies Online Course (eMB), with coaching reinforcement provided by participants’ home visitors. Control participants received usual HV services. Primary outcomes were depressive and anxiety symptoms, and perceived stress. Outcomes were measured at baseline, one-week post-intervention, and three months post-intervention.

**Results:**

Thirty-three individuals were randomized to eMB with coaching and nine to usual HV. Participants were on average 28 years old, racially and ethnically diverse, and had a modal education attainment of less than a Bachelor’s degree. Mean and modal number of eMB lessons completed were 3.61 (2.97SD) and 7, with mean and modal completed coaching sessions 4.32 (3.31SD) and 3, respectively. No main effects of time or treatment group were found, nor were any treatment by time interactions statistically significant. Follow-up analyses revealed a main effect of time on the GAD-7 and CES-D, and dosage analyses found a significant time by intervention dose interaction on the GAD-7, with eMB participants receiving a lower dose exhibiting greater anxiety. Baseline depressive symptoms were positively associated with the number of eMB lessons completed.

**Conclusion:**

Future research should prioritize strategies to facilitate eMB lesson completion given findings suggesting greater dosage may lead to improved anxiety outcomes. Home visitors’ relationships with perinatal clients should be leveraged to promote eMB lesson completion.

**Trial registration:**

The study was registered with Clinicaltrials.gov (NCT05714956, registration date: January 25, 2023)

## Introduction

Postpartum depression is the most common health complication associated with pregnancy, with an estimated 10–19% of new mothers meeting criteria for a depressive episode (O’Hara & McCabe, 2013; Shorey et al., 2018). The consequences of postpartum depression are manifold. Individuals with postpartum depression are at increased risk for future depressive episodes and are likely to experience negative social and physical functioning (O’Hara & McCabe, 2013; Slomian et al., 2019; Wisner et al., 2004). The effects of postpartum depression extend across generations, with depressed mothers exhibit lower parenting self-efficacy, less responsiveness to their child’s cues, and less positive emotion when engaging with their children compared to non-depressed mothers (Barnes & Theule, 2019; Ferber et al., 2008; Herrera et al., 2004; Kohlhoff & Barnett, 2013).

Effective interventions to prevent postpartum depression have been developed, with a United States Preventive Services Taskforce Report (USPSTF) concluding that pregnant and postpartum individuals at risk for postpartum depression should be referred to counseling interventions (O’Connor et al., 2019). The Mothers and Babies Course (MB) was specifically called out by the USPSTF as one of the two most effective interventions for preventing postpartum depression, with a series of randomized controlled trials (RCTs) demonstrating MB’s impact on reducing maternal depressive symptoms (effect sizes from −0.28 to −0.73) and prevention of new cases of major depression. MB was initially developed as a group-based intervention (Muñoz et al., 2007), with recent adaptation developing an individualized (“1-on-1”) version that has also demonstrated effectiveness (Tandon et al., 2018; Tandon et al., 2022).

The Mothers and Babies Online Course (eMB; (Barrera et al., 2015)), a web adaptation of the MB, was designed to expand the reach of evidence-based prevention of postpartum depression interventions to at-risk birthing individuals from low-resourced and Spanish-speaking communities. The structure and framework are guided by the 8-week MB group approach (Le et al., 2011) with delivery of core theoretical constructs via text pages, graphical images, brief videos, and guided audio relaxation exercises. The eMB content is enhanced by digital worksheets whereby users can personalize and download typed responses to instructional prompts. With the intent to reduce access barriers, the eMB was designed as a fully-automated digital intervention available to perinatal individuals regardless of depression status, medical insurance, or service delivery affiliations (Barrera et al., 2022). A benefit of the open access approach was the global availability to those seeking guidance to address changes in mood during and after pregnancy (Barrera et al., 2014) and the limited human resources needed beyond the pre-programmed emails and tailored prompts.

Despite the emerging evidence demonstrating the effectiveness of prevention and treatment of postpartum depression digital interventions (e.g., online, text message, app-based; (Lewkowitz et al., 2024; Mu et al., 2021)), they are relatively nascent and, therefore, were not included in the USPSTF report as a recommended intervention type for at-risk perinatal women. Critical is the fact that evidence on the effectiveness of online interventions, specifically, varies because of methodological and implementation differences. Further, online interventions that are not self-guided, like the eMB, lead to lower user engagement and higher attrition rates (Mu et al., 2021). In contrast, digital interventions that incorporate a human touch (e.g., trained clinicians, peers) lead to stronger empirical evidence and greater user satisfaction (Mohr et al., 2011). To date, however, guided online interventions for the prevention of postpartum depression have not been examined using home visitors as the human factor.

Home visiting (HV) programs serve perinatal individuals across the United States, with most states combining local/state and federal Maternal, Infant, and Early Childhood Home Visiting (MIECHV) funding. Although most evidence-based HV programs address stress and mental health in their curricula, most HV agencies struggle to meet the needs of families exhibiting postpartum depression and are actively seeking solutions to better respond to the prevalence of postpartum depression experienced by the families they serve (Ammerman et al., 2010; Tandon et al., 2020). Several previous trials have found the group and 1-on-1 modalities of MB to be effective in reducing depressive symptoms and perceived stress among women enrolled in HV programs. While in-person MB modalities are effective, intervention dosage among participants is highly variable with multiple studies finding increased effects on depressive symptoms and perceived stress among individuals receiving more MB lessons (Le et al., 2011; Tandon et al., 2022).

This study aimed to test whether an online modality (eMB) could be feasibly implemented and improve participant’s mental health. We previously described the coaching protocol developed to accompany HV participants’ receipt of eMB (Baez et al., 2024). This article describes findings from a pilot RCT examining eMB dosage received, delivery of coaching sessions by home visitors, and intervention effectiveness when delivered via evidence-based HV programs. Additional mixed-methods implementation outcomes will be presented in a separate manuscript.

## Methods

### Study Design

This study employed a randomized controlled trial (RCT) design to examine the implementation and preliminary effectiveness of the Mothers and Babies Online Course (eMB) intervention, when integrated into existing home visiting (HV) programs. Given our RCT design, this study adheres to CONSORT guidelines. Eligible participants were randomized in a 4:1 allocation ratio to either the intervention group (eMB) or the control group (usual HV care). Randomization was conducted via the randomization module in REDCap. Unequal distribution with more participants randomized to the intervention condition was done to allow for more implementation data related to intervention feasibility and acceptability. All research activities were conducted in accordance with the Declaration of Helsinki and were approved by the Institutional Review Boards of Northwestern University (STU00216763) and Palo Alto University. Informed consent was obtained from all study participants prior to any data collection.

### Recruitment and Study Participants

Participants were recruited from April 2022 to December 2023 from HV agencies across the U.S., inclusive of urban, suburban, and rural areas. Home visitors employed at HV agencies were provided with study brochures and flyers which were shared with eligible clients. Clients could opt to have their contact information shared with the study team or reach out independently. Study staff contacted interested individuals by phone, text, or email and followed up with a phone call to explain the study and assess interest. A secure REDCap link was sent to interested participants, directing them to an online consent form followed by a screening questionnaire. Consent could alternatively be conducted by phone. Screening was used to determine eligibility based on perinatal depression risk. Those deemed ineligible were thanked and compensated $10 for their time. Eligible participants providing informed consent subsequently completed a baseline assessment.

**Figure 1.**
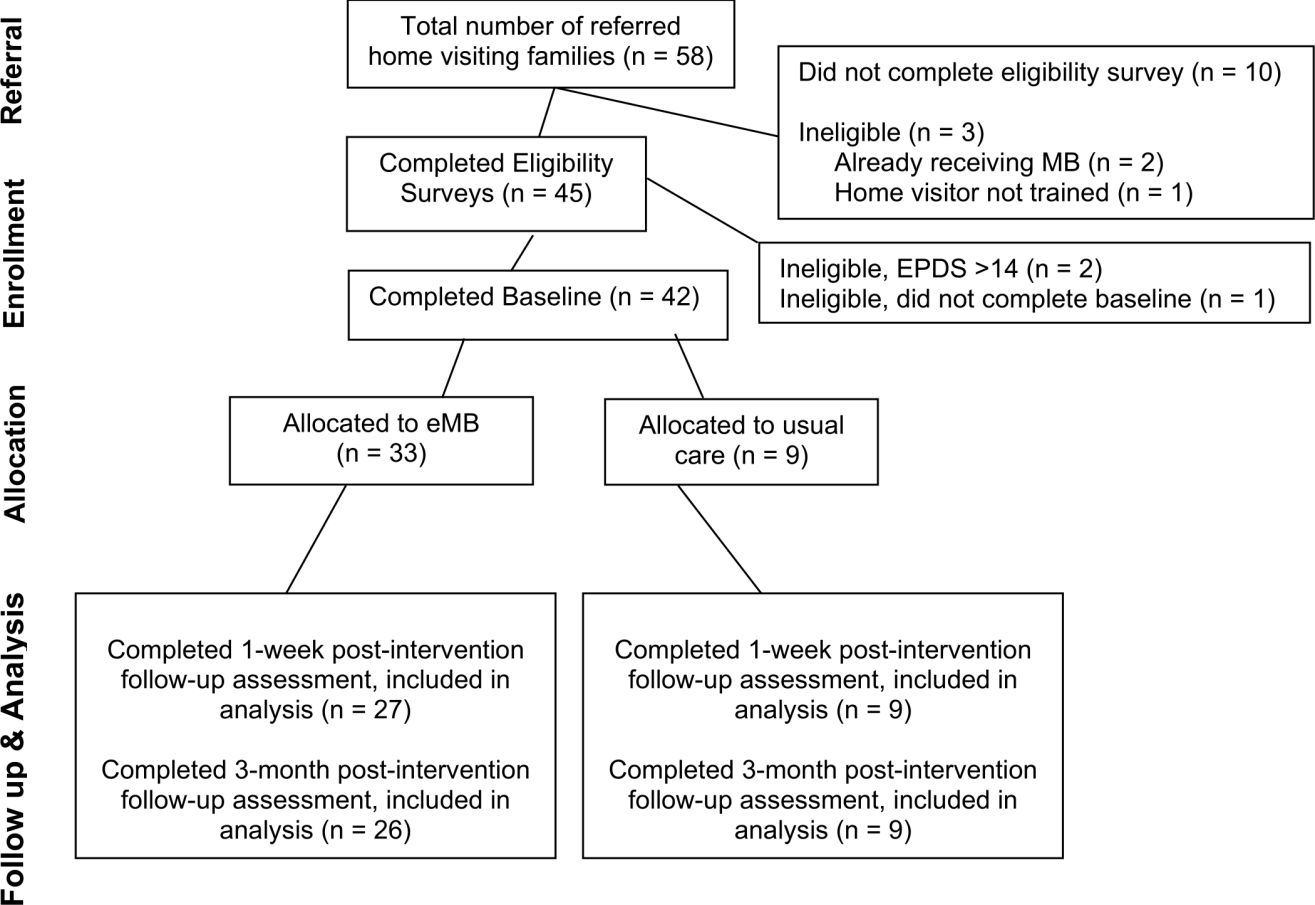
CONSORT flow diagram showing participant flow through each stage of the RCT

Eligibility criteria for parent participants included: enrolled in HV, age 16 or older, English proficiency, currently pregnant or parenting an infant under 12 months, access to internet and an electronic device (phone, laptop, tablet, etc.), and risk for perinatal depression defined by at least one of the following: Edinburgh Postnatal Depression Scale (EPDS) score 5–14 (Cox et al., 1987), Postpartum Depression Predictors Inventory-Revised (PDPI-R) (Beck, 2002) score ≥ 3.5 (prenatal) or ≥ 4.5 (postnatal), or self-reported personal depression. A total of N = 42 perinatal individuals were recruited. Participants were randomized to the intervention (n = 33) or control group (n = 9) by a study coordinator using a computer generated 4:1 allocation ratio (Fig. 1). Sample size was determined using generally accepted guidelines for pilot studies, with an emphasis on enrolling a greater proportion of participants in the intervention arm to obtain more data on eMB implementation.

Home visitors of participating clients were recruited and trained to serve as eMB coaches. HVs completed their own consent process and were eligible to participate if they referred a client and completed training in the eMB coaching protocol. Twenty-five home visitors enrolled in the study and referred at least one client.

### Data Collection Procedures and Measures

#### RCT Participants.

All RCT participants completed self-report questionnaires at three time points:baseline, one-week post-intervention, and three-months post-intervention. Data collection occurred via REDCap or, by request, phone interviews. Our primary intervention effectiveness outcomes were depressive symptoms, anxiety symptoms, and perceived stress. Depressive symptoms were assessed using the Center for Epidemiologic Studies-Depression Scale (CES-D) (Radloff, 1977)—a 20-item self-report checklist that measures existence and severity of depressive symptoms consistent with DSM symptom criteria. Anxiety symptoms were assessed using the Generalized Anxiety Scale-7 item version (GAD-7) (Spitzer et al., 2006), which measures existence and severity of anxiety symptoms. Perceived stress was assessed using the Perceived Stress Scale-4-item version (Cohen et al., 1983), a self-report checklist that asks participants to assess perceptions of recent stress. We assess intervention engagement by measuring the number of eMB lessons completed by a participant.

#### Home Visitors.

Home visitors completed the following data collection activities: (a) baseline demographic assessment, (b) post-intervention REDCap survey, and (c) weekly assessments to assess eMB coaching while clients completed the eMB intervention. The weekly assessments were used to calculate the number of eMB coaching sessions provided by a home visitor.

### Intervention Conditions

#### eMB.

Participants randomized to the intervention arm received access to eMB, an online, self-guided version of the Mothers and Babies intervention (Barrera et al., 2015). eMB consists of seven lessons (Table 1), each introducing cognitive-behavioral skills aimed at promoting mood management, delivered through a mix of written content, audio/video clips, interactive worksheets, and reflective activities. Participants were encouraged to complete lessons weekly or biweekly but retained access throughout the study regardless of completion rate. An optional eighth lesson provided guided audio recorded relaxation activities that participants could subsequently use to manage their mood.

Home visitors were notified when a client enrolled in the study and was randomized to the eMB condition. Home visitors received web-based training from a member of the research team on the coaching protocol developed for this study by the investigators and community collaborators (Baez et al., 2024). Once a client completed an eMB lesson, an email notification was sent to the study team who then forwarded the email to the client’s home visitor. This notification was a prompt for the home visitor to complete a eMB coaching session, developed to last about 10–15 minutes. Each coaching session focused on: (a) encouraging engagement in subsequent eMB lessons, (b) helping clients apply eMB lesson content to their daily lives, and (c) addressing barriers to eMB lesson completion.

#### Usual HV.

Participants randomized to the control group continued receiving standard services from the HV agency where they were enrolled. Usual care typically includes prenatal and parenting support, early childhood development education, linkage to health care, and referrals to additional services. Control participants did not receive access to the eMB intervention during the study but were offered access following study completion.

### Data Analysis

Descriptive statistics were used to calculate the number of eMB lessons attended by individuals randomized to the intervention arm (range 0–7), as well as the number of coaching sessions (range 0–7) completed by home visitors. Analyses for maternal mental health outcomes (depression, anxiety, perceived stress) used a two-way mixed-effects analysis of variance (ANOVA) across the three study time points. Follow-up analysis was conducted among just intervention participants to evaluate the effect of intervention dose on each mental health outcome, with dosage divided into low (0–3 lessons) and high (4–7 lessons) categories. Post-hoc tests were corrected for multiple comparisons using the Bonferroni method. Correlation analyses were performed to examine the relationship between baseline depressive symptomatology and number of eMB lessons completed.

## Results

### Sample Demographics

A demographic summary of the study sample, stratified by intervention arm, is provided in Table 2. Mothers were on average 28 years old in both the eMB and control groups. More than half of eMB (58%) participants were partnered, while less than a quarter (33%) of control participants were in a relationship. Both groups were racially and ethnically diverse. Approximately half (52%) of eMB participants were White, a quarter (24%) were Black, and 21% were Hispanic. About two-thirds of control participants (78%) were White and one third (33%) was Hispanic. Intervention group participants were more likely to have achieved more than a high school degree/GED and were also more likely to also have a child.

The mean number of eMB lessons completed was 3.61 (2.97 SD), with the mode being completion of all seven lessons (33% of participants) (Table 3). Home visitors completed an average of 4.32 (3.31SD) coaching sessions, with the provision of three coaching sessions found to be the mode. All home visitors with clients receiving eMB completed at least one coaching session.

**Figure 2:**
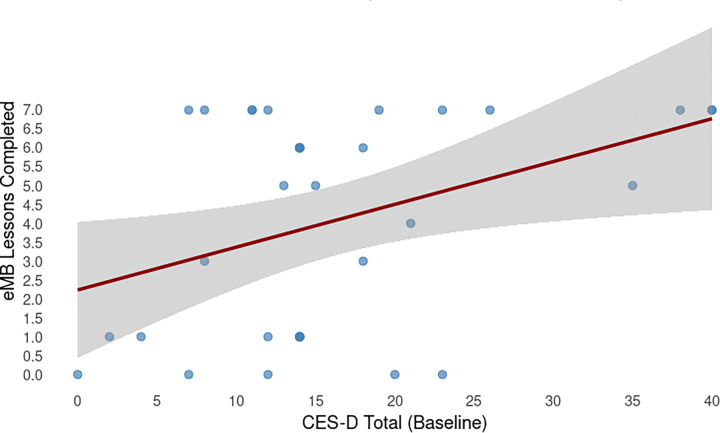
Association Between Baseline Depressive Symptoms and Lesson Completion

Mean (SD) scores on the GAD-7, CES-D, and PSS at each timepoint are summarized in Table 3. Results revealed no main effects of time or treatment group, and no significant treatment group by time interaction on each mental health outcome. Directionality of change in depressive symptoms was in the intended direction, with follow-up analyses revealing a main effect of time on the CES-D [*F*(2,42) = 3.88, *p* = .029]. Dosage analyses found a significant time by intervention dose interaction on the GAD-7, with individuals receiving a low intervention dose exhibiting significantly higher GAD-7 scores at one-week post-intervention (b = 2.59, *p* = .036) and three-month follow-up (b = 2.76, *p* = .028) compared to baseline, with p-values corrected for multiple comparison (Table 4). Prior to correction, the CES-D scores showed a similar pattern at 1-week post-intervention, with individuals on average reporting fewer depression symptoms with a higher treatment dose (b = −1.51, *p* = .046); however, this post hoc analysis did not survive multiple comparison correction (*p* > .05). There was no interaction between treatment dose and CES-D scores at 3-months (b = −.12, *p* = .86) or PSS scores at 1-week (b = −.76, *p* = .15) or 3-months (b = −.01, *p* = .97) post-intervention. There was a positive association between baseline depressive symptoms and the number of eMB lessons completed (*r* = .41, *p* = .02) (Fig. 2).

## Discussion

This study adds to the growing literature on digital health interventions aimed at the prevention or treatment of postpartum depression and anxiety (Lewkowitz et al., 2024; Mu et al., 2021). While previous meta-analyses found small yet significant findings suggesting that digital health interventions are effective in addressing postpartum depression and anxiety, our pilot study yielded mixed results. No overall intervention effects were found on our key mental health outcomes, although significant reductions were seen over time in depressive symptoms among intervention participants and greater reductions in anxiety symptoms were found among those receiving more eMB lessons. Caution should be used in comparing our findings with previous digital health intervention studies addressing postpartum depression or anxiety, as earlier studies were characterized by significant heterogeneity regarding participant demographics, measurement tools and timelines, and intervention content.

Findings from this pilot study were consistent with the mixed results reported in prior research examining online prevention interventions for perinatal depression and anxiety (Nguyen & Pengpid, 2025). Although there is growing evidence supporting the effectiveness of online interventions for improving symptoms of perinatal depression and anxiety, variability in intervention characteristics, including theoretical approach (e.g., cognitive behavioral therapy vs. mindfulness-based approaches), timing of delivery (during pregnancy vs. postpartum), and level of intervention intensity (e.g., weekly vs. monthly; guided vs. unguided) complicates comparisons across studies and limits conclusions regarding their collective impact on effectiveness outcomes (Duan et al., 2025). Nevertheless, several intervention characteristics emerge as noteworthy.

It is well established that digital interventions incorporating human support (e.g., therapists) are associated with greater engagement and stronger outcomes, including in the context of postpartum depression (Evans et al., 2022; Pan et al., 2025). To the best of our knowledge, this study is the first to deliver a digital health intervention aimed at preventing depression and anxiety within the context of HV programs, with home visitors serving as coaches to guide participants’ use of the eMB intervention. Home visitors serving as coaches may represent a more scalable form of human support compared to licensed therapists, as this model can be more readily integrated into existing workflows and may enhance long-term sustainability by reducing reliance on highly trained professionals. This approach is further supported by recent evidence demonstrating minimal differences in effectiveness when digital interventions are delivered by clinicians versus trained non-clinician professionals (Savoia et al., 2025). Given home visitors’ established relationships and familiarity with their clients, they may be particularly well positioned to personalize guidance related to eMB use and to help mitigate external stressors disproportionately experienced by high-risk populations such as low socioeconomic status, single parenting, and exposure to racism which are psychosocial stressors shown to contribute to lower engagement and reduced intervention effectiveness (Nguyen & Pengpid, 2025, Silang et al., 2022).

Pilot studies such as the one described herein should primarily focus on examining logistical aspects that can inform future study design (Kistin & Silverstein, 2015). As such, pilot studies are not designed to be adequately powered to detect statistically significant differences within or across study groups. Specific to this study, although we did not detect effects of eMB when comparing outcomes between intervention and control group participants over time, there appears to be a signal of improvement in depressive symptoms over time among intervention recipients as well as greater reductions in anxiety symptoms among eMB participants receiving a greater dose of the intervention. Perceived stress stayed largely consistent over time among eMB recipients, which runs contrary to recent studies examining the in-person MB intervention (Tandon et al., 2022; Tandon et al., 2025). This could be a reflection that our sample had more chronic stressors than participants in previous trials. Almost all eMB participants had other children, which could also contribute to higher stress levels. It is also possible that the online modality with home visiting coaching was less effective in encouraging intervention participants to practice some of the stress reduction activities throughout the intervention that would be expected to lead to reductions in perceived stress.

Our pilot study was useful in several regards in directing future research and intervention delivery. Although the modal session completion was seven sessions (i.e., the full eMB intervention), we found variability in eMB lessons completed, suggesting that intervention participants may need automated prompts from the eMB platform and/or additional prompts from their home visitors to facilitate ongoing engagement with eMB. Coaching sessions also varied across home visitors. While this could be a function of home visitors’ non-adherence to the coaching protocol, all home visitors completed at least one coaching session suggesting that other reasons are likely also driving coaching variability. It is possible that some home visitors asked clients whether they wanted or needed a coaching session and only provided a coaching session if time permitted during a home visit, a reflection that even though coaching sessions were designed to take only 10–15 minutes, home visitors have many competing demands placed on them during a scheduled home visit.

In this study, we did not exclude individuals with depressive symptomatology at baseline that exceeded a cutoff typically indicative of postpartum depression. When examining the relationship between baseline mental health status and eMB lesson completion, we found that individuals with greater baseline depressive symptoms completed more eMB lessons. This finding is notable, as eMB and the MB intervention on which it is based have been used almost exclusively as preventive interventions among pregnant individuals, but findings from our pilot suggest that individuals with greater baseline symptomatology may be appropriate candidates for eMB. This could be, in part, due to the relative ease of the self-guided eMB platform among individuals with more significant depressive symptoms that may hinder their ability to effectively engage with scheduled, synchronous therapeutic sessions. This result mirrors earlier findings from Barrera and colleagues (2022) who also found that more depressed individuals engaged with eMB at a higher rate, suggesting that eMB could potentially be useful for individuals already exhibiting perinatal depression—a population that is different from the focus of previous MB trials that were preventive in focus.

Study investigators have increasingly been approached by HV agencies and systems across the United States to receive training on the in-person MB intervention. This is due, in part, to MIECHV requirements that require HV programs to provide referrals to evidence-based mental health services for clients exhibiting elevated depressive symptoms (Health Resources & Services Administration, 2026). While future research should be conducted to obtain more robust evidence of eMB effectiveness when implemented in HV, eMB represents a potentially low-resource alternative to synchronous MB intervention delivery that may be preferred by some clients and home visitors. To help inform future implementation of eMB, we are currently conducting a preference trial in which HV clients are asked to select either eMB or the 9-session synchronous version of MB delivered by a home visitor. We anticipate the preference trial will build on findings from the current pilot study—for example, should individuals with higher baseline depressive symptoms gravitate toward the self-guided eMB modality, that would complement the findings herein that more depressed individuals engaged in more eMB lessons.

## Study Strengths and Limitations

Digital health interventions have been shown to be more effective when accompanied by human support. However, the way human support is delivered is likely to influence its effectiveness. As described in Baez et al. (2024), we employed user-centered design techniques that elicited HV staff perspectives to develop the coaching protocol used by home visitors in this study. A key recommendation from the user-centered design work was to align the structure of the coaching sessions with the way home visitors check-in with families about other goals and activities. Another strength of this study is the potential for scaling eMB throughout HV networks across the United States. Caution should be taken, however, in advocating for scaling until stronger evidence of eMB effectiveness can be generated. As noted above, it appears that HV clients with higher baseline symptoms were more likely to engage in eMB, suggesting that this may be a useful tool for HV programs needing an approach to serve pregnant individuals and new parents exhibiting elevated depressive symptoms. Many HV programs struggle to meet the mental health needs of their families due to limited mental health resources external to their agencies and have reached out for training on synchronous delivery of Mothers and Babies. Synchronous delivery of Mothers and Babies is still likely to be the primary modality for intervention delivery in HV, given the frequent and ongoing contact that home visitors have with perinatal individuals that facilitates completion of MB lessons. However, synchronous delivery can still be challenging in rural environments and for families with multiple caregiving and work responsibilities, opening an opportunity for eMB to fill an important gap.

As a pilot study, this study is not aimed at generating conclusive statements about eMB effectiveness. With a larger sample size, it is possible that the same magnitude of change in the two study groups would have been statistically significant. As it stands, results presented in this manuscript, juxtaposed with eMB acceptability and feasibility data (Barrera et al., 2022; Hamil et al., 2025) and considerable HV agency interest in integrating mental health interventions, suggest that future adequately powered clinical trials are warranted to further examine eMB effectiveness.

## Conclusions

Rates of depression among perinatal populations have stayed constant over time, with recent years suggesting potential increased prevalence (Khadka et al., 2024). As home visiting and other early childhood providers outside of the traditional mental health system are increasingly charged with meeting the mental health needs of their families, there is a concurrent need for accessible, low-cost, and evidence-informed interventions such as eMB. Strategies to promote greater engagement among families and home visitors with eMB should be developed, which is likely to lead to more widespread improvements in mental health symptoms among pregnant individuals and new mothers receiving the intervention.

## Supplementary Material

Supplementary Files

This is a list of supplementary files associated with this preprint. Click to download.

• Table234.docx

## Figures and Tables

**Table 1:** Titles and Descriptions of eMB Lessons.

#	Lesson Title	Description
1	Purpose & Overview	Review online course format, intervention theoretical principles, and general instructions on website use
2	Thoughts & My Mood	Educational material describing the relationship between cognitions and emotions
3	Fighting Harmful & Increasing Helpful Thoughts	Application of skills to teach and reinforce principles of Thoughts & Mood lesson
4	Activities & My Mood	Educational material describing the relationship between behaviors and pleasant activities, and emotions
5	Pleasant Activities Help Make a Healthy Reality	Application of skills to teach and reinforce principles of Activities & Mood lesson
6	Contact with Others & My Mood	Educational material and application of skills describing the impact of relationship on emotions
7	Planning for the Future & Graduation	Course overview and summary
8 (optional)	Guided Relaxation Exercises	Brief guided audio recordings tailored for pregnant and postpartum individuals

## Data Availability

The datasets used and/or analyzed during the current study are available from the corresponding author on reasonable request.
